# *Phytobacter* spp: the emergence of a new genus of healthcare-associated Enterobacterales encoding carbapenemases in Argentina: a case series

**DOI:** 10.1016/j.infpip.2024.100379

**Published:** 2024-06-10

**Authors:** Marisa Almuzara, Roxana Cittadini, Germán Traglia, María Sol Haim, Denise De Belder, Carla Alvarez, Zandra de Lourdes Reynal O'Connor, Cecilia Vera Ocampo, Claudia Barberis, Mónica Prieto, Josefina Campos, Carlos Vay

**Affiliations:** a Universidad de Buenos Aires, Facultad de Farmacia y Bioquímica, Departamento de Bioquímica Clínica, Cátedra Microbiología Clínica, Hospital de Clínicas “José de San Martín”, CABA, Argentina; b Sanatorio Mater Dei, CABA, Argentina; c Unidad de Genómica y Bioinformática, Departamento de Ciencias Biológicas, CENUR Salto, Universidad de La República, Uruguay; d Unidad Operativa Centro Nacional de Genómica y Bioinformática ANLIS "Dr Carlos G. Malbrán", CABA, Argentina; e Laboratorio de Bacteriología Especial, ANLIS "Dr Carlos G. Malbrán", CABA, Argentina

**Keywords:** *Phytobacter*, Enterobacterales, Carbapenemases, Severe infection, Identification

## Abstract

Members of the genus *Phytobacter* (order *Enterobacterales*) are isolated from the natural environment and clinical settings. Identification of *Phytobacter* strains based on biochemical characteristics is complicated due to taxonomic confusion, and they are often misidentified by automated identification systems in laboratories.

In this study we describe the first three clinical cases associated with *Phytobacter* spp. reported in Argentina. We describe the identification, the molecular analysis using whole genome sequencing and the potential clinical relevance.

## Introduction

Members of the genus *Phytobacter* (order *Enterobacterales*) may be isolated from both the natural environment and from clinical settings [[1], [2], [3]]. They are recognised as plant growth-promoting rhizobacteria helping plants grow. However, in recent times they are increasingly reported in clinical infections such as bacteraemia such as cases in Brazil resulting from contaminated total parenteral nutrition [4].

*Phytobacter* genus includes four published species [5].

*Phytobacter diazotrophicus,* was originally described by Zhang *et al.* [2] based on few endophytic nitrogen-fixing bacteria isolated from surface-sterilised stems and roots of wild rice (*Oryza rufipogon*) in Hainan, China. Later, Pillonetto *et al.* emended the description of the genus *Phytobacter* and its type species *Phytobacter diazotrophicus* and described a new species *Phytobacter ursingii* sp.nov. [1].

In 2020, two other species were assigned to *Phytobacter* genus. Ma *et al.* proposed the unification of the genus *Metakosakonia* and the genus *Phytobacter* to a single genus *Phytobacter* and reclassified *Metakosakonia massiliensis* as *Phytobacter massiliensis* comb. nov. [6]. Madhaiyan *et al.* describe *Phytobacter palmae*, a new species isolated as an endophyte from surface-sterilised leaflet tissues of oil palm (*Elaeis guineensis*) from Singapore [7].

The identification of Phytobacter strains based on biochemical characteristics is complicated due to taxonomic confusion, and they are often misidentified by automated identification systems in laboratories [1].

Smits *et al.* have further unravelled the taxonomic confusion of the genus *Phytobacter* using modern molecular tools based on whole-genome sequencing analysis with digital DNA-DNA hybridization and average nucleotide identities (ANI), assigning isolates previously characterised as *Pantoea agglomerans* into two distinct species: *Phytobacter diazotrophicus* and *Phytobacter ursingii* [3].

In the present study we describe the first three clinical cases in Argentina in which *Phytobacter* spp. were isolated from clinical samples.

## Case 1

An 85-year-old female with breast cancer and lung metastasis undergoing chemotherapy was admitted with symptoms of fever, syncopal episode, dyspnoea, and a productive cough. She had a history of previous hospitalisations. The clinical interpretation of her presentation was that the symptoms were likely to represent hospital-acquired pneumonia. She was treated with piperacillin-tazobactam for 7 days and had a good clinical response.

From a rectal swab, which had been sent for screening for carbapenemase-producing Entrobacterales (CPE), an Enterobacterium grew which was identified by whole genome sequencing (WGS) as *Phytobacter diazotrophicus.* Three acquired resistance genes were detected in this organism: *bla*_SHV-12_, *bla*_KPC-2_, located in a transposon (Tn4401) and *bla*_qnrE_, responsible for resistance to quinolones.

## Case 2

A 26-day-old female premature neonate with a birth weight of 1000g, had been born following an extra-membranous pregnancy which occurred from week 19 of the pregnancy. She had required advanced cardiopulmonary resuscitation (endotracheal tube placement, chest compressions, and administration of adrenaline) and mechanical respiratory assistance for 14 days. She had also been treated with UV light therapy for 3 days due to neonatal jaundice. At birth, blood cultures were collected which subsequently had no growth. She completed antibiotic treatment with intravenous ampicillin for 7 days and gentamicin for two days, because of slow clinical progress. She had received parenteral nutrition since the first day of life for 14 days. On the 13th day following birth, she decompensated clinically and haemodynamically. Blood cultures were collected (2 samples; BACTEC® Peds Plus Medium). Gram-negative bacilli subsequently identified as *Phytobacter ursingii* resistant to ampicillin only, grew in both samples after 7 hours' incubation. She had received further treatment with ampicillin and gentamicin but did not respond to treatment and died.

## Case 3

A 78-year-old man patient, with a history of benign prostatic hyperplasia, presented with fever and chills 10 days after a prostatectomy. Urine and blood cultures were collected. The urine analysis showed 2–4 leukocytes/HPF, >20 erythrocyte/HPF, 0–1 epithelial cells/HPF.

*Phytobacter diazotrophicus* identified by WGS and which was resistant to ampicillin was isolated from the urine (with a colony count greater than 100,000 CFU (colony forming units)/ml and from the blood cultures. The BACTEC® Lytic/10 Anaerobic and BACTEC Plus Aerobic blood cultures were positive, after 18 and 28 hours' incubation respectively. The clinical presentation was interpreted as sepsis with a likely urinary source. The patient was treated with piperacillin-tazobactam and ciprofloxacin with a good clinical response.

**Figure 1:**
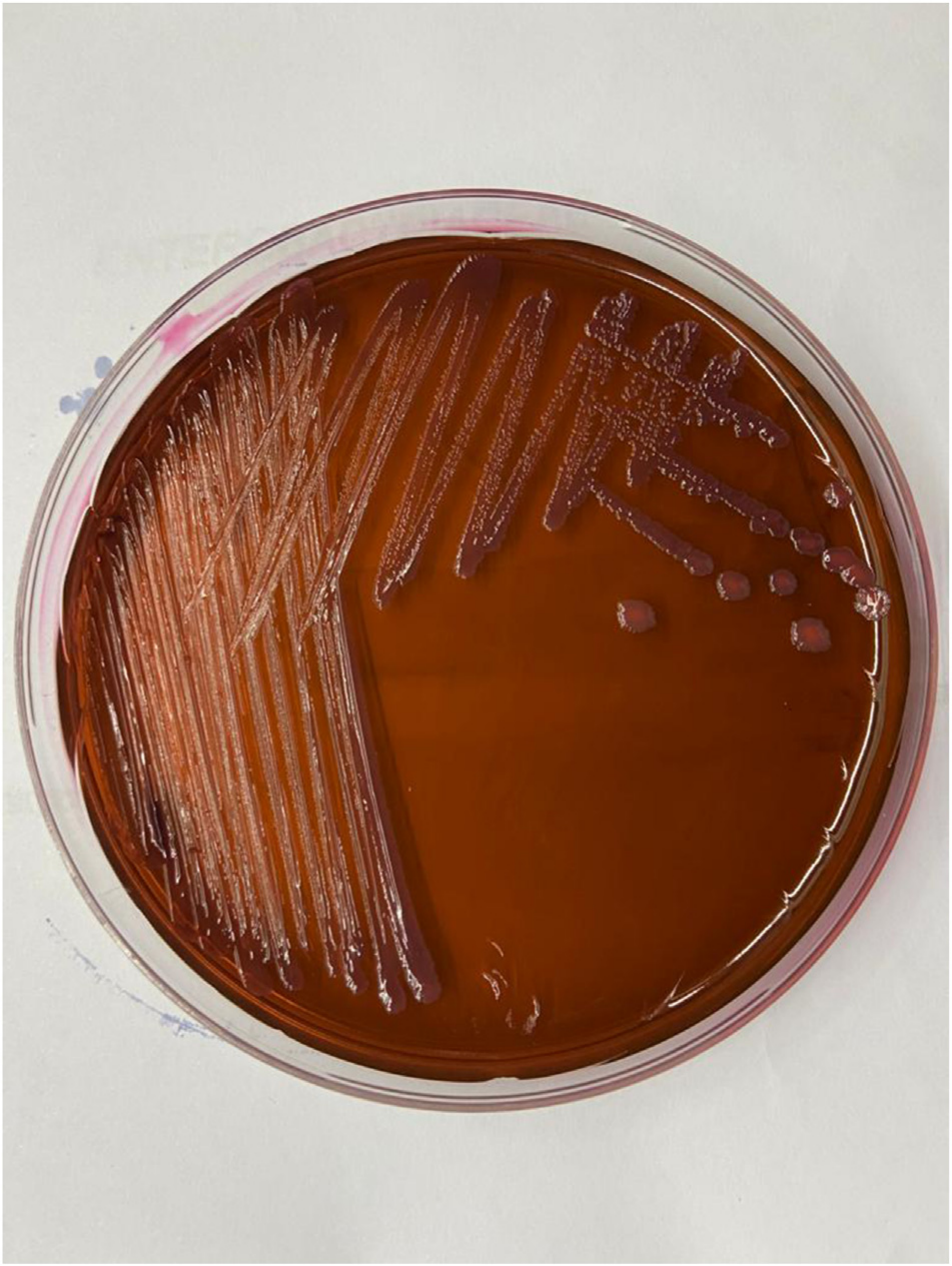
Colonies of a *Phytobacter* isolate on EMB Levine.

In all three cases, the colonies on nutrient agar were nonpigmented and on EMB Levine Agar (Difco ™) the organisms produced mucoid lactose-fermenting colonies (Figure 1).

The phenotypic identification of the isolates was initially carried out with conventional biochemical tests. Using this methodology, identification of the 3 isolates was compatible with non-pigmented, indole producing *Pantoea* spp. (Table I)

**Table I:** Characteristics of the isolates (cases 1 to 3) and *Pantoea agglomerans*, *Phytobacter diazotrophicus and Phytobacter ursingii*

Characteristic	1	2	3	*P. diazotrophicus*^a^	*P. ursingii*^a^	*P.agglomerans* ATCC 27155^T^
Colour	−	−	−	−	−	Yellow
Growth at 37°C	+	+	+	+	+	+
Motility	+	+	+	+	+	+
Urease	−	−	−	−	−	−
Lysine decarboxylase	−	−	−	−	−	−
Ornithine decarboxylase	−	−	−	−	−	−
Arginine dehydrolase	−	−	−	−	−	−
Indole production	+	+	+	−	−	+
β-glucuronidase	−	−	−	unk	unk	unk
β galactosidase	+	+	+	+	+	+
Voges-Proskauer	+	+	+	+	+	+
Aesculin	+	+	+	+	+	+
Malonate	+	+	+	+	+	+
DNA hydrolysis	−	−	−	−	−	+
Gelatinase	−	−	−	−	−	+
**Acid from**						
Glucose	+	+	+	+	+	+
Lactose	+	+	+	+	+	n/v
Saccharose	+	+	+	+	+	+
Adonitol	−	−	−	−	−	−
Mannitol	+	+	+	+	+	+
Maltose	+	+	+	unk	unk	+
Mannose	+	+	+	+	+	+
Xylose	+	+	+	+	+	+
Inositol	−	−	−	−	−	−
Rhamnose	+	+	+	+	+	+
Glycerol	+	+	+	+	+	+
Raffinose	−	−	−	−	−	−
Trehalose	+	+	+	+	+	+
Melibiose	−	−	−	−	−	−
Galactose	+	+	+	+	+	+
Arabinose	+	+	+	+	+	+
Cellobiose	+	+	+	+	+	n/v
Sorbose	−	+	−	+	−	−

+ positive; − negative; n/v weak reaction; unk-unknown.

a Smits *et al.* [3].

Identification by the use of an automated Vitek 2 ® compact system (bioMérieux,Marcy-l’Étoile, France) using a GN Colorimetric Identification Card yielded respectively, the following biocodes: 601714453500010 (Unidentified); 607730457500000 (*Pantoea* spp. 95 %), and 607734557500050 (*Pantoea* spp. 89 %).

Species identification was also carried out by using matrix-assisted laser desorption ionization–time of flight mass spectrometry (MALDI-TOF MS (Bruker ® Daltonics) and the current Bruker Daltonics database (MBT-BDAL-12.0 MSP library); identification by this methodology yielded *P. ursingii* (case 2) and *Phytobacte*r sp. (cases 1 and 3).

To confirm the identification of our isolates, WGS was performed using the Illumina MiSeq sequencer at the National Center for Genomics and Bioinformatics ANLIS "Dr Carlos G Malbrán", Buenos Aires, Argentina. The assembly of the sequence was carried out using SPAdes Assembler (version.3.1.0). Whole genomes were deposited at DDBJ/EMBL/GenBank under the accession no. JAWJAA000000000 (*P. diazotrophicus* CVMA1), JAWJAB000000000 (*P. diazotrophicus* CVMA24) and JAWJAC000000000 (*P. ursingii* CVMA36).

The sequenced genomes were annotated with the Prokka [8] and Roary software [9] was used to align the core genes. The SNP-sites program was used to identify polymorphisms (SNPs) [10], to then carry out the phylogenetic study using IQTREE2 under the GTR model with the gamma distribution (GTR+GAMMA) predicted by ModelFinder [11].To carry out the analysis of the genomes, various bioinformatics tools were used. Species identification by genome sequence were performed by ANI% and DNA-DNA hybridization in silico using ani.rb script [12] and GGDC software [13], respectively. Likewise, the determination of the resistome and virulome was carried out with BLAST (E-value<10^−5^) and different databases such as MegaRes [14] and VFDB database [15].

ANI% analysis revealed that CVMA1 (case 1) and CVMA24 (case 3) were identified as *Phytobacter diazotrophicus* (with a 98% genomic similarity to *Phytobacter diazotrophicus* NRB043), whereas CVMA36 (case 2) was identified as *Phytobacter ursingii* (with a 99% genomic similarity to *Phytobacter ursingii* ATCC 27989). Furthermore, DNA-DNA hybridization in silico analysis yielded results consistent with ANI% findings. CVMA1 and CVMA24 were acknowledged as *Phytobacter diazotrophicus*, while CVMA36 was reconfirmed as *Phytobacter ursingii*.

Antimicrobial resistance gene identification was conducted using the MegaRes database. *Phytobacter diazotrophicus* CVMA1 contains the *bla*_*KPC-2*_, *bla*_*SHV-12*_ genes (harbored within the Tn4401 transposon), and *qnrE1* genes, responsible for resistance to quinolones. We also identified a homologous gene to fosA (Amino Acid Identity: 75.31%; Coverage: 95.95%) in all the sequenced isolates, which could explain the fosfomycin resistance phenotype. Virulence gene identification was carried out using the VFDB database. In *Phytobacter* genomes, 68, 69, and 70 virulence genes were identified in CVMA1, CVMA24, and CVMA36, respectively (Supplementary Table S1).

The antibiotic susceptibility tests were performed using the VITEK® 2 system with the AST-079 (GNS susceptibility card) panel. The MIC breakpoints used in this study were those established by the Clinical and Laboratory Standards Institute [16] for *Enterobacterales* (Table II). In addition, fosfomycin susceptibility was determined using a 200 μg disk test in agar Mueller Hinton. CMVA1 and CMVA 24 showed no halo (6mm) and CMVA36 showed a small halo (15mm), therefore all the isolates were resistant.

**Table II:** Antibiotic susceptibility (MIC^a^ μg/ml) of *Phytobacter* spp. isolates

Antibiotic	Case 1	Case 2	Case 3
Ampicillin	≥ 32	16 (I)	16 (I)
Ampicillin/Sulbactam	≥ 32	≤ 2	≤ 2
Piperacillin/Tazobactam	≥ 128	≤ 4	≤ 4
			
Cefalexin	≥ 64	≤ 4	≤ 4
Cefotaxime	≥ 64	≤ 1	≤ 1
Ceftazidime	≥ 64	≤ 1	≤ 1
Cefepime	8	≤ 1	≤ 1
Imipenem	≥ 16	≤ 0.25	≤ 0.25
Meropenem	≥ 16	≤ 0.25	≤ 0.25
Amikacin	4	≤ 2	≤ 2
Gentamicin	≤ 1	≤ 1	≤ 1
Ciprofloxacin	0.5	≤ 0.25	≤ 0.25
Nitrofurantoin	128	64	128
Colistin	≤ 0.5	≤ 0.5	≤ 0.5
Trimethoprim/Sulfamethoxazole	≤ 1.0	≤ 1.0	≤ 1.0

a MIC: minimum inhibitory concentration.

## Discussion

The colonial morphology of *Phytobacter* spp. on EMB Levine agar, resembles *E. coli* and *Citrobacter* since it is a strong lactose-fermenting Gram-negative bacillus. However, it can also be lactose non-fermenting and have a colonial morphology that resembles *Enterobacter cloacae* complex. Given that the biochemical tests for *Phytobacter* spp include negative results for lysine decarboxylase, ornithine decarboxylase and arginine dihydrolase, it can be misidentified as *Pantoea* spp. or *Pantoea agglomerans* by means of conventional biochemical tests or, by automated identification systems, as has been previously pointed out by other authors [4,17] potentially resulting in the overestimation of the role of this latter species in human infection [4,18].

Likewise, the identification by means of mass spectrometry systems (including MALDI-TOF MS, Vitek®-MS and MALDI Biotyper-Bruker Microflex®) can also be incorrect if the database is not updated [2].

Although *Phytobacter* has been described in human samples by Pillonetto M *et al.* [1] only recently whole genome sequencing has shown that the genus *Phytobacter* has been incorrectly classified as other species since the 1970s. [3].

Its clinical relevance has been demonstrated in studies, including 24 isolates studied by Smits *et al.* [3] in a period of 5 years (2016–2021) with more than 50% recovered from blood cultures. It has also been implicated in outbreaks worldwide including in Brazil and the United States. [4].

Many of the cases identified as having *Phytobacter* spp infection were sepsis after receiving intravenous fluids or the use of medical devices, often in neonatal intensive care units [4].

The finding of multi-resistant strains of *Phytobacter* spp has been reported by other authors [2,12,19,20]. One of our isolates was multi-resistant (with 2 acquired resistance genes for beta-lactam antibiotics: *bla*_SHV_-_12_, and *bla*_KPC-2_, located in a transposon (Tn4401)), demonstrating its ability to become a reservoir of resistance genes in the hospital environment. Every isolate showed resistance to fosfomycin. The phenotype could be explained by the presence of a homologous gene of fosA.

## Conclusions

Although there are only several reported cases of *Phytobacter* infections, its true clinical relevance could be hidden due to the phenotypic misidentification and the lack of reference organisms in MALDI-TOF MS databases. The emergence of new genera of *Enterobacterales* has posed challenges for the diagnosis and treatment of related infections. In this sense, since sequencing approaches might not be available for many laboratories, the correct identification of *Phytobacter* spp. remains a challenge for routine clinical microbiology diagnostics.

## Ethics statement

All procedures performed in this study met the ethical standards of Hospital de Clínicas Jose de San Martín, Buenos Aires, Argentina, and the 1964 Declaration of Helsinki and further amendments. Consent to publish has been obtained from the patients.

## Conflict of interest

None.

## Author contributions

MA, RC and CV collected the data and performed phenotypic identification; CB wrote the manuscript; GT, MSH, JC contributed with genomic identification. All authors participated actively in the interpretation of the microbiological results and approved the final version of the manuscript.

## Funding

This work was supported by grants from the Secretaría de Ciencia y Técnica de la 10.13039/501100005363Universidad de Buenos Aires (UBACyT project number 20020170100109BA) to C. V.
